# Pre-dopa Deep Brain Stimulation: Is Early Deep Brain Stimulation Able to Modify the Natural Course of Parkinson’s Disease?

**DOI:** 10.3389/fnins.2020.00492

**Published:** 2020-06-05

**Authors:** Mauro Porta, Domenico Servello, Edvin Zekaj, Gabriel Gonzalez-Escamilla, Sergiu Groppa

**Affiliations:** ^1^Center for Movement Disorders and Tourette Syndrome, Galeazzi Hospital, Milan, Italy; ^2^Functional Neurosurgery Unit, Galeazzi Hospital, Milan, Italy; ^3^Movement Disorders and Neurostimulation, Department of Neurology, University Medical Center of the Johannes Gutenberg University, Mainz, Germany

**Keywords:** Parkinson’s disease, deep brain stimulation, subthalamic nucleus, early deep brain stimulation, early intervention

## Abstract

Deep brain stimulation (DBS) is an established therapy for the management of Parkinson’s disease (PD). However, DBS is indicated as the disease progresses and motor complications derived from pharmacological therapy arise. Here, we evaluate the potential of DBS prior to levodopa (L-Dopa) in improving quality of life (QoL), challenging the state of the art for DBS therapy. We present data on clinical manifestation, decision finding during early indication to DBS, and trajectories after DBS. We further discuss current paradigms for DBS and hypothesize on possible mechanisms. Six patients, between 50 and 67 years old, presenting at least 5 years of PD symptoms, and without L-Dopa therapy initiation, received subthalamic nucleus (STN) DBS implantation. In the six PD cases, indication for DBS was not driven by motor complications, as supported by current guidelines, but by relevant QoL impairment and patient’s reluctance to initiate L-Dopa treatment. All patients treated with STN-DBS prior to L-Dopa presented improvement in motor and non-motor symptoms and significant QoL improvement. All patients reduced the intake of dopamine agonists, and five are currently free from L-Dopa medication, with no reported adverse events. We introduce a multicenter observational study to investigate whether early DBS treatment may affect the natural course of PD. Early application of DBS instead of L-Dopa administration could have a pathophysiological basis and be prompted by a significant incline on QoL through disease progression; however, the clinical value of this proposed paradigm shift should be addressed in clinical trials aimed at modulating the natural course of PD.

## Introduction

The treatment of Parkinson’s disease (PD) patients should aim to control both motor and non-motor symptoms to maintain optimal functioning in daily-life activities while preventing further motor complications and minimizing the risk of long-term side effects, therefore positively modifying the natural course of disease.

Levodopa (L-Dopa) therapy is the most common and efficient pharmacological solution to control motor symptoms; however, notorious complications such as motor fluctuations, dyskinesia, and wearing-offs develop, significantly impacting the quality of life (QoL) (Ray Chaudhuri et al., 2018). Treatment strategies including higher L-Dopa dosages (daily doses of 600 mg and above), stepwise dosage (increasing dose according to motor symptoms) of L-Dopa, or dopamine agonists, are often limiting because of the insufficient control of the motor symptoms and negative effects on daily-life activities.

Stereotactic treatment for PD with deep brain stimulation (DBS) of the subthalamic nucleus (STN) or the globus pallidus internus (GPi) is a highly efficient, evidence-based therapy to alleviate motor symptoms and is safe and well tolerated in patients at different disease stages (Fenoy and Simpson, 2014; Engel et al., 2018; Kim et al., 2018). Growing body of evidence suggests that DBS further improves non-motor symptoms (Kurtis et al., 2017) and QoL. Thus, it may hold the potential to modify the disease course of a neurodegenerative disorder, but current insufficient information about this possibility exists. To date, no evidence-based pharmacological paradigms for this exists; however, several strategies have shown to influence the disease course; a timely decision about the initiation of the next therapeutic step is an important component (Schuepbach et al., 2013; Charles et al., 2014; Hacker et al., 2018; Lhommee et al., 2018). However, DBS is currently indicated at later disease stages, when the pharmacological strategies are not sufficient for controlling motor complications (dyskinesia, fluctuations, and wearing-off) or if the patient has motor symptoms that do not respond sufficiently to standard oral treatments (i.e., refractory rest tremor).

Previous studies have pointed to the possible positive effects of DBS at earlier disease stages (Schuepbach et al., 2013; Charles et al., 2014; Suarez-Cedeno et al., 2017; Hacker et al., 2018; Lhommee et al., 2018), whereas only one case report exists about the possibility to apply DBS without L-Dopa-induced effects (Servello et al., 2016). We extend current existing information to present a multicenter case series on PD patients for whom STN-DBS therapy was initiated as a treatment option at an earlier stage than in current clinical practice (i.e., following L-Dopa therapy).

We advocate that decision-taking for DBS initiation without L-Dopa based on functional impairment without a good control on dopamine agonist therapy could represent more than a point of inflection to modify the natural disease course but also reflect a timely strategy to modify the brain network activity in vulnerable circuits toward the physiological range. Shifting the intervention to slow disease progression should deliver timely and wider therapeutic windows that offer the maximum impact on brain function. Improvement of QoL, social, and occupational functioning may be granted by slowing clinical deterioration and neural degeneration over the longer range. We finally discuss on the feasibility of this therapy pathway and provide possible pathophysiological explanations for the ability of DBS to modify the PD natural course.

## Materials and Methods

This case series consists of six right-handed PD patients (two female; mean age 59.3 ± 7.1 years) treated with DBS without L-Dopa administration and treated at the Galeazzi Hospital Movement Disorders Center of Milan (Italy) and at the Section of Movement Disorders and Neurostimulation of the Johannes Gutenberg University Mainz (Germany).

All patients reported being aware of the association of higher L-Dopa doses with dyskinesia, wearing-off, and further medication-induced motor complications. Therefore, the patients refused to initiate L-Dopa therapy. All patients presented motor-symptom worsening over time with a subjective impairment of QoL. The inclusion criteria further included clinical diagnosis of idiopathic PD [according to MDS Criteria (Postuma et al., 2015, 2018): with classical parkinsonian tremor at rest or dyskinesias in one extremity]; Hoehn and Yahr (H&Y) stage ≤2.0 in the best ON condition; presence of fluctuations and/or dyskinesias; 39-Item Parkinson’s Disease Questionnaire (PDQ-39) completed; at least 5 years of PD symptoms at the time of surgery; and one of the two following forms of impairment: (i) impairment on daily activities (Schwab) due to PD symptoms despite medical treatment or (ii) impairment of social adaptation (measured with a modified SOFAS-scale) due to PD symptoms despite medical treatment (>50%).

The exclusion criteria included major depression with suicidal thoughts (Beck Depression Inventory >25) (earlier episodes of MD are not excluded); dementia (Mattis score ≤132); acute psychosis (benign hallucinations or earlier psychotic episodes are not excluded); need for nursing care; any medical or psychological problems that may interfere with a smooth conduction of the study protocol (e.g., cancer with a limited life expectancy); illiteracy; drug or alcohol addiction; surgical contraindications; fertile women not using adequate contraceptive methods; women who are pregnant or breastfeeding; and simultaneous participation in another clinical treatment trial.

Clinical diagnosis and follow-up visits were conducted by neurologists trained in movement disorders. The study involved collection of demographic data, disease onset and duration, PD clinical features regarding both motor and non-motor symptoms and pharmacological management, and DBS parameters along with any adverse events related to surgery and stimulation.

### Levodopa Challenge

A L-Dopa/dopaminergic challenge test was performed on all potential DBS candidates. Here, participants were given a supra-threshold dosage (L-Dopa) of 120–150% of the morning L-Dopa equivalent dose (LED). The percentage improvement, obtained by comparing the Unified Parkinson’s Disease Rating Scale Part III (UPDRS-III) in the “OFF” medication (med-OFF) and “ON” medication (med-ON) conditions, was used to determine a patient’s L-Dopa response (motor improvement following L-Dopa administration).

### Surgical Procedures

The surgical procedure has been previously described (Groppa et al., 2014; Muthuraman et al., 2017; Koirala et al., 2018). All patients underwent stereotaxic DBS surgery under local anesthesia on the basis of the patient’s individual anatomy, provided by brain MRI, and microelectrode recording to localize bilateral STN. Two patients were implanted with directional leads and pulse generators (Abbott St. Jude Medical Infinity), whereas the rest of patients were implanted with omnidirectional electrodes (model 3389 DBS, Medtronic) and pulse generators (Activa PC, biphase stimulation).

Postoperatively, initial stimulation parameters were pulse width of 60 ms, frequency of 130 Hz, and amplitude of 1.5 V. These parameters were progressively adjusted in each patient. DBS-lead locations were visually confirmed with a postoperative brain CT scan.

Clinical improvement (in percentage) for STN-DBS was measured by comparing the UPDRS-III scores med-OFF condition and the ON DBS OFF medication (stim-ON) condition.

### Statistical Analyses

Categorical variables, including PDQ-39 and H&Y, were compared using paired *t*-tests. Repeated-measures ANOVA was used to compare UPDRS-III scores across all conditions (med-OFF, L-Dopa challenge, and two follow-ups after DBS).

## Results

Clinical details of each patient before STN-DBS and outcome UPDRS-III scores are given in Table 1. Overall, the study patients, treated with STN-DBS without L-Dopa treatment initiation, improved both motor and non-motor symptoms. All six patients reduced the intake of dopamine agonists. Table 2 shows details of medical management for each reported case before and after DBS implantation.

**TABLE 1:** Case series of Parkinson’s disease (PD) patients with implanted STN-DBS without initiation of L-Dopa therapy.

	**Age at STN-DBS (years)**	**Sex**	**Age at disease onset (years)**	**Onset symptoms**	**Subsequent developed symptoms**	**L-Dopa challenge UPDRS-III improvement**	**Neuropsycho- logical assessment**	**Max. follow-up**	**Follow-up UPDRS-III score (med-OFF/stim-ON condition)**
Case 1	53	Male	47	Right-sided resting tremor, bradykinesia, and rigidity. DaT-SPECT was positive for DaT depletion, with rostral–caudal decrease in tracer uptake, maximally affecting the left posterior dorsal striatum	Freezing of gait symptoms, very mild bilateral motor fluctuations, and right hemibody dystonic cramps	41% (from 17 to 10)	Unremarkable	9 years	8 increasing
Case 2	66	Male	61	Bilateral bradykinesia. DaT-SPECT was positive for DaT depletion, showing a bilateral rostral-caudal decrease in tracer uptake, maximally affecting the left posterior dorsal striatum	Gait initiation symptoms, freezing of gait, and refractory bradykinesia affecting both arms	66% (from 27 to 9)	Minor deficits of working memory and verbal fluency	2 years	8 stable
Case 3	50	Male	44	Left shoulder and left arm discomfort with subsequent rigidity and debilitating resting tremor. DaT-SPECT was positive for DaT depletion, showing a rostral-caudal decrease in tracer uptake, maximally affecting the right posterior dorsal striatum	Left hemibody bradykinesia with painful cramps during night time	66% (from 18 to 6)	Unremarkable	2 years	6 stable
Case 4	63	Female	58	Bilateral bradykinesia and rigidity, worse in the upper left limb, showing also a mild resting tremor	Mild motor fluctuations, pain, and dystonic cramps in both legs	94% (from 19 to 1)	Unremarkable	6 months	1 stable
Case 5	67	Male	57	Right hemibody rigidity and resting tremor, worse in his upper limb	Gait initiation issues and bradykinesia	55% (from 42 to 19)	Unremarkable	6 months	17 stable
Case 6	57	Female	46	Bradykinesia and a mild rest tremor	Freezing of gait symptoms and rigor	42% (from 26 to 15)	Borderline scores on STM and EF	6 months	9 stable

*1-Dopa, levodopa; DaT, dopamine transporter; SPECT, single-photon emission computed tomography; UPDRS-III, Unified Parkinson’s Disease Rating Scale Part III; STM, short-term memory; EF, executive function; STN, subthalamic nucleus; DBS, deep brain stimulation.*

**TABLE 2:** H&Y stage and quality-of-life scores.

	**H&Y**		**PDQ-39**		**LED**	
	**Before DBS**	**After DBS**	**Before DBS**	**After DBS**	**Before DBS**	**After DBS**
Case 1	2	1	31	19	180	140
Case 2	2.5	1.5	44	22	157	52
Case 3	1.5	1	42	18	445	0
Case 4	2	1.5	30	18	450	0
Case 5	2.5	1.5	45	24	52	0
Case 6	2.5	1.5	44	26	362	257

*Clinical data before DBS was measured during OFF medication condition. H&Y, Hoehn & Yahr staging; PDQ-39, the 39-Item Parkinson’s Disease Questionnaire; LED, levodopa equivalent doses in milligrams (mg); DBS, deep brain stimulation.*

In all patients, DBS therapy was activated 3 weeks after surgery, and no stimulation-related side effects were recorded.

**FIGURE 1:**
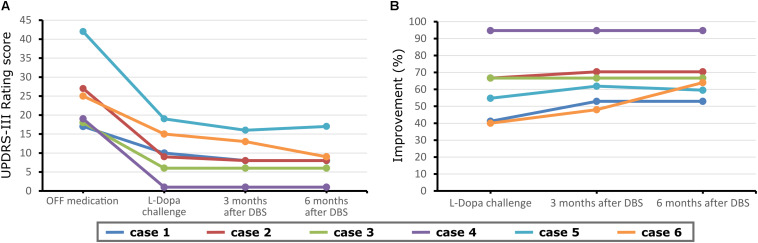
Unified Parkinson’s Disease Rating Scale Part III (UPDRS-III) scores before and at follow-up (3 months) after deep brain stimulation (DBS) surgery for each case reported and at a further proximal follow-up (6 months). **(A)** Motor scores in the medication OFF condition before DBS implantation and also stimulation OFF after DBS implantation. **(B)** Motor scores in the medication ON before DBS implantation, and medication OFF/stimulation ON condition after electrode implantation.

**FIGURE 2:**
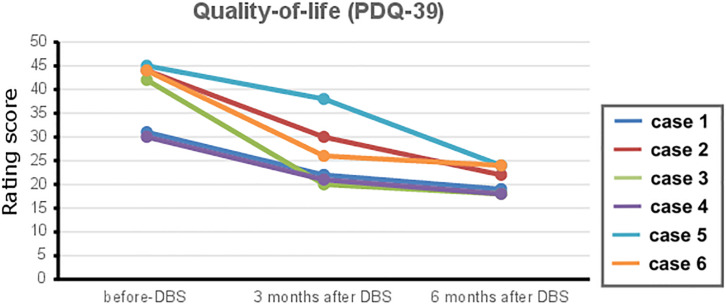
The 39-Item Parkinson’s Disease Questionnaire (PDQ-39) outcome scores of quality of life before and after deep brain stimulation (DBS) therapy (follow-up at 3 and 6 months) after DBS surgery for each case reported. Reduced PDQ-39 scores translate to an improvement in quality of life in Parkinson’s disease (PD) patients.

No adverse events regarding surgery, stimulation issues, or non-motor symptoms of disease have been reported by the time of this report. The repeated-measures ANOVA revealed a significant change in the UPDRS-III scores [*p* = 0.001, *F*(3,20) = 8.1], for differences between med-OFF and L-Dopa challenge (*p* = 0.0009, *T* = 6) and effects of DBS in comparison with med-OFF (*p* = 0.0004, *T* = 7.2) and to L-Dopa challenge (*p* = 0.02, *T* = 2.7). UPDRS-motor scores assessed in the DBS clinics at L-Dopa challenge improved an average of 60.7% (range 40–94.7%), whereas after DBS implantation, UPDRS-III improved 66% on average, spanning from 48–94.7% (Figure 1), and stable during follow-up periods ranging from 3 months to 9 years. QoL outcomes (Table 3), such as the PDQ-39, which are considered integral measures for well-being, and patients’ own evaluation of the impact of the disease also improved considerably (*p* = 0.0003, *T* = 5.1; Figure 2). The comparison for the H&Y scores revealed also a significant improvement after DBS implantation (*p* = 0.038, *T* = 2.36).

**TABLE 3:** STN-DBS parameters.

	**Left hemisphere**			**Right hemisphere**		
	**Amplitude (V)**	**Frequency (Hz)**	**Width (ms)**	**Amplitude (V)**	**Frequency (Hz)**	**Width (ms)**
Case 1	3.1	200	60	2.5	200	60
Case 2	3	130	60	3	130	60
Case 3	2.5	130	60	3.2	200	60
Case 4	3.2	216	60	3.2	80	60
Case 5	3.5	216	90	2.5	130	60
Case 6	3.6	130	60	2.3	130	60

*STN, subthalamic nucleus; DBS, deep brain stimulation.*

## Discussion

DBS has become the standard therapy for PD patients with treatment-refractory motor symptoms such as fluctuations and dyskinesias, with the potential to considerably improve non-motor symptoms and life quality at the long term. We postulate the potential of DBS to modify the disease course by switching the time point of implantation to even earlier disease stages, when considerable functional impairment and a QoL dip occur, but no secondary effects have been induced by prolonged L-Dopa usage. Although with different clinical manifestations, the patients described has similar outcome features: good preoperative response to L-Dopa challenge, improvement of PD motor symptoms, reduction of pharmacological therapy, and greater QoL up to 9 years after therapy initiation. Thus, better and longer-lasting protective effects of STN-DBS in PD patients are assumed.

Despite profound improvements, the current clinical procedure for recommending DBS has severe limitations. First, patients who undergo implantation are in advanced disease stages and have motor complications. At this stage, not only an abnormal brain networks functioning persisted over a long period, but also severe physical and psychosocial disabilities have developed. Secondly, at the currently indicated time point, DBS intervention mainly improves dopa-responsive symptoms, leaving further motor and non-motor symptoms not sufficiently influenced. Disturbances in cognition, vegetative symptoms, and other complaints most likely resulting from an abnormal functioning of the brain circuits are also insufficiently treated within the conventional DBS framework. Therefore, the remaining open question is whether DBS treatment administered at even earlier disease stages is able to enhance the history-modifying potential of DBS in a clinical settings (Servello et al., 2016).

Our reported patients presented a disease duration of at least 5 years, which is similar to the current average encountered in other studies (Groiss et al., 2009). Based on the existing animal models and human data suggesting that DBS may modify the course of PD (Kahn et al., 2012; Ngoga et al., 2014; Musacchio et al., 2017), a novel hypothetical shift in the clinical management is to apply DBS at earlier disease stages (Kahn et al., 2012; deSouza et al., 2013; Charles et al., 2014; Servello et al., 2016; Suarez-Cedeno et al., 2017; Hacker et al., 2018). In PD patients, DBS has been recently linked to longer survival (deSouza et al., 2013). However, in that study, it is not clear if a disease course-modifying effect was present through modulation of non-motor symptoms or if results were led by an improved motor function. Early DBS seem to positively influence tremor severity and grant long-term advantage in comparison with a later implantation (Hacker et al., 2018). Similarly, timely DBS application to the appearance of motor complications is shown to significantly improve QoL and motor symptoms in comparison with the best-medical treatment group (EARLYSTIM) (Lhommee et al., 2018). Similarly, early DBS is related to less perioperative complications and to a more uncomplicated postoperative course and more effective rehabilitation (Allert et al., 2018). Together, these data underline our approach that instead of applying DBS in disease stages with motor complications, in which neurodegeneration leading to a disruption and malfunctioning of brain networks is advanced, we rather evaluate the disease course-modifying potential of DBS in patients at earlier disease stages. We propose that this framework has the capability to positively influence brain circuits and induce long-term improvements in both motor and non-motor symptoms.

In 2013, the EARLYSTIM trial focused on DBS feasibility in populations at early disease stages, defined as short disease duration (7.3 ± 3.1 years) and motor complications (for 3 years or less) (Schuepbach et al., 2013). This trial showed that earlier DBS is superior to medical treatment alone, resulting in a longer and more stable QoL improvement. While in the EARLYSTIM, the mean QoL improvement after STN-DBS was ∼27% (7.8 points) in comparison with baseline PDQ-39 measures, and the QoL improvement in our cases represented about 46% (18.2 points).

Both studies are also concordant with other previous findings from the Vanderbilt group of a pilot STN-DBS study conducted among 30 patients, aged 50–75 years, with a very short duration of idiopathic PD (>6 months but <2 years), an H&Y Stage II in off-medication state, and without motor fluctuations or dyskinesias (Charles et al., 2014). However, inclusion of patients with a disease duration of less than 5 years may harbor the risk of including cases with atypical parkinsonism (Suarez-Cedeno et al., 2017), which may explain the lack of significant improvement in the QoL in their study.

When comparing STN-DBS and L-Dopa brain connectivity effects, similar clinical effects are observed but with different network modulation within the motor system (Mueller et al., 2018). Hence, we proposed that STN-DBS before L-Dopa therapy could further promote a longer-lasting physiological and psychosocial functioning and positively modulate the neurodegenerative process. This hypothesis finds support on recent reports on the potential of DBS to shift the network response toward the physiological range (Kim et al., 2015; Horn et al., 2019), whereas DBS outcomes have been constantly related to the integrity of widespread brain regions connected to the stimulation sites in PD (Muthuraman et al., 2017; Koirala et al., 2018; Irmen et al., 2020) and in other diseases (Gonzalez-Escamilla et al., 2019). Thus, an effect on the neurodegenerative side is also expected.

In our report, the decision to perform the DBS was driven by the influence of patients’ QoL (our main outcome) through the motor and non-motor symptoms, which was clearly improved by STN-DBS without L-Dopa treatment. However, unlike the general PD population treated with DBS at a mid/late stage, our patients had no motor fluctuations or dyskinesias induced by prolonged L-Dopa usage; the patients reduced the intake of dopamine agonists, are currently free from L-Dopa medication, and report no adverse events. From a pathophysiological standpoint, this earlier intervention over the disease networks could further lead to a shift of the brain functioning toward the physiological range, thus influencing the course of neurodegeneration. The potential of DBS to modify the clinical course of PD is related to its ability to alleviate motor and non-motor symptoms, permitting the patients to maintain mobility and remain more flexible, slowing functional deterioration and avoiding complications, allowing the patients longer time to remain engaged in their daily activities, their relationships, and social environment, which are reflected as improved QoL.

DBS has been widely demonstrated to help PD patients in coping with motor and non-motor symptoms as the disease progresses (Benabid et al., 1994; Okun and Foote, 2010; Fasano et al., 2012). Many researchers have perceived that DBS may affect the course of disease through different mechanisms of action, including influence cell survival and preservation of neural network function. DBS works by electrically modulating neuronal firing rates, influencing the basal ganglia aberrant activity, and increasing blood flow. Therefore, a pronounced effect of STN-DBS on plasticity in the basal ganglia circuitry likely affecting disease symptoms is possible (Spieles-Engemann et al., 2011; Musacchio et al., 2017), whereas maintenance of synaptic integrity at the DBS target and the connected cortex as well as preservation of subcortical–cortical networking might explain the positive effects of DBS (Horn et al., 2017).

Studies focusing on morphological or functional brain network integrity and reservoir to disease, together with clinical trajectories, may help to elucidate more on this topic. Connectivity studies focusing on the network between the STN and brain cortex, including the supplementary and motor cortices, the superior frontal gyrus, and the cerebellum, show that functional and structural connections correlate with clinical improvement after DBS. Suggesting that connectivity of the stimulation site to the distributed network is an important mediator of DBS responses (Vanegas-Arroyave et al., 2016; Akram et al., 2017; Horn et al., 2017) and that the therapeutic benefit of DBS may depend on modulation of remote brain regions connected to the stimulation site (Muthuraman et al., 2017; Koirala et al., 2018). Of notice, although all subjects presented reduced LED, in two of them, less marked responses were registered, which could be attributed to the stimulation parameters and/or to the DBS electrode locations. However, these two patients also presented the lowest responses during the L-Dopa challenge. Thus, these effects in combination with DBS recommendation criteria should be taken into account in future patient trials testing the extension of the use of DBS in PD patients without L-Dopa therapy.

Despite all current findings and as discussed through the manuscript, information on whether or not DBS can modify the neurodegenerative course of the disorder is currently limited; a targeted approach addressing vulnerable networks might be favorable. Because most of the actual treatments could not modify the disease course, when applied in the motor phase of the disease, early interventions are needed. Accordingly, our work proposes to consider the application of the DBS in the early disease stages when first functional impairments arise, thus having the potential for long-term modulation of the symptoms and life quality, directly or indirectly modifying the disease course. One of the patients, previously described in Servello et al. (2016), initiated L-Dopa treatment after 9 years of DBS; therefore, no further follow-up information was provided. Another limitation of the study is the variable disease duration of the included patients (ranging from 5–11 years). A next step would be to limit the disease duration to a shorter time to ensure that patients are in the early stages of PD. However, the courses of the diseases are rather heterogeneous in PD patients. Here, robust biomarkers should be developed.

Further supported by consistent reports about patient safety at different disease stages and low risk for complications after DBS (Fenoy and Simpson, 2014; Engel et al., 2018; Kim et al., 2018), we propose that future clinical trials should test the extension of the use of DBS in patients with early PD without motor complications and L-Dopa response but symptom-related QoL impairment. This work could be of major importance for addressing disease course-modifying therapies in PD treatment. The cases presented here seem to support the hypothesis that early DBS treatment positively modifies the course of PD, possibly delaying the development of motor and non-motor complications enhanced by high-dose L-Dopa therapy. If further quantitative studies can confirm that an even earlier implantation drive the functional and QoL maintenance through modification of the disease symptoms, this would implicate a novel approach driven by physiological network functioning and not by clinical or phenomenological decisions, as taken now by the assessment of motor fluctuations. Such findings would have a profound effect on clinical practice and change the treatment algorithms for this condition. So far, in our cases, no psychosocial disability or interruptions in family, social, or professional activities, which would lead to maladjustment or indirect disease progression, have developed. Thus, we believe that patients could truly benefit longer from this safe and effective intervention that is already available.

## Conclusion

This observational study evidences that DBS treatment exerts beneficial effects on motor symptoms and quality of life in early stages of PD, if applied when first functional and QoL-impairments occur, even before L-Dopa treatment initiation. These novel data and paradigm shift proposal challenge current algorithms for PD treatment and grants further studies evaluating the disease course-modulating potential of very early DBS application in larger populations.

## Data Availability Statement

All datasets generated for this study are included in the article/supplementary material.

## Ethics Statement

Ethical review and approval was not required for the study on human participants in accordance with the local legislation and institutional requirements. The patients/participants provided their written informed consent to participate in this study. Written informed consent was obtained from the individual(s) for the publication of any potentially identifiable images or data included in this article. All patients signed forms consenting the clinic to store and analyze their data, tissue, and blood samples for scientific purposes and submission of the report to the journal.

## Author Contributions

MP and SG conceptualized, organized, and executed the research project, and reviewed and critiqued the manuscript. DS conceptualized and executed the research project, and reviewed and critiqued the manuscript. EZ executed the research project and reviewed and critiqued the manuscript. GG-E conceptualized, organized, and executed the research project, wrote the first draft, and reviewed and critiqued the manuscript.

## Conflict of Interest

The authors declare that the research was conducted in the absence of any commercial or financial relationships that could be construed as a potential conflict of interest.
